# Pathology of Hereditary Breast and Ovarian Cancer

**DOI:** 10.3389/fonc.2020.531790

**Published:** 2020-09-29

**Authors:** Anjelica Hodgson, Gulisa Turashvili

**Affiliations:** ^1^Department of Laboratory Medicine and Pathobiology, University of Toronto, Toronto, ON, Canada; ^2^Department of Pathology and Laboratory Medicine, Mount Sinai Hospital, Toronto, ON, Canada

**Keywords:** BRCA, hereditary breast cancer, hereditary tubo-ovarian cancer, high-grade serous carcinoma, triple-negative breast cancer

## Abstract

Hereditary breast and ovarian cancer (HBOC) syndrome is most commonly characterized by deleterious germline mutations in *BRCA1* and *BRCA2*. HBOC patients are prone to the development of malignant neoplasms in multiple organs including the breast, ovary, and fallopian tube. From a pathological perspective, a number of morphological features have been described in *BRCA*-associated breast and tubo-ovarian cancers. For example, breast cancers diagnosed in *BRCA1*-mutation carriers are frequently of a high Nottingham grade and display medullary morphology and a triple-negative and/or a basal-like immunophenotype. In contrast, breast cancers in *BRCA2*-mutation carriers are similar to sporadic luminal-type tumors that are positive for hormone receptors and lack expression of human epidermal growth factor receptor 2. Cancers arising in the fallopian tube and ovary are almost exclusively of a high-grade serous histotype with frequent Solid, pseudo-Endometrioid, and Transitional cell carcinoma-like morphology (“SET features”), marked nuclear atypia, high mitotic index, abundant tumor infiltrating lymphocytes, and necrosis. In addition, pushing or infiltrative micropapillary patterns of invasion have been described in *BRCA*-associated metastases of tubo-ovarian high-grade serous carcinomas. Besides *BRCA1* and *BRCA2* mutations, alterations in a number of other homologous recombination genes with moderate penetrance, including *PALB2, RAD51C, RAD51D, BRIP1*, and others, have also been described in HBOC patients with varying frequency; however, distinct morphological characteristics of these tumors have not been well characterized to date. In this review, the above pathological features are discussed in detail and a focus is placed on how accurate pathologic interpretation plays an important role in allowing HBOC patients to receive the best possible management.

## Introduction

Hereditary breast and ovarian cancer (HBOC) is a genetic tumor syndrome most commonly caused by germline deleterious mutations in *BRCA1* and *BRCA2.* The *BRCA1* and *BRCA2* tumor suppressor genes (chromosome 17q21 and 13q12.3, respectively) (1–6) encode for proteins involved in DNA double strand break repair by homologous recombination, one of the critical maintenance mechanisms of DNA integrity (7). In order to complete this function, the BRCA proteins interact with a host of other molecules which together form a protein complex; without a functional BRCA complex, the cell relies on alternative mechanisms for DNA repair, some of which are error prone and may further contribute to the development of genetic aberrations (8). Because of this phenomenon, HBOC patients with germline *BRCA1* and *BRCA2* mutations have an increased risk for the development of a number of neoplasms, particularly those arising in the breast as well as ovary and fallopian tube (9) (hereby referred to as “tubo-ovarian cancer”).

In the general population, the risk for the development of breast and tubo-ovarian cancer is approximately 10–15% and 1–2%, respectively. In *BRCA1* and *BRCA2* mutation carriers, the risk increases to approximately 45–65% and 20–50%, respectively (10–14). Germline mutations in other homologous recombination genes including *BARD1, BRIP1, PALB2, RAD51C, RAD51D*, and others (all encoding proteins involved in BRCA protein stability and/or function), have also been identified to varying degrees in breast and tubo-ovarian cancer patients. Studies evaluating the lifetime risk of disease development in these patients have estimated a range of at least 15–35% for breast cancer (15, 16) and 5–10% for tubo-ovarian cancer (17–20). Mutations in some of these genes impart an increased risk for either breast or tubo-ovarian cancer with minimal to no increased risk for the development of the other tumor type (i.e., increased risk of breast cancer without risk of tubo-ovarian cancer, and vice versa) (21–24). For example, *BRIP1, RAD51C*, and *RAD51D* mutation carriers have an increased risk for tubo-ovarian cancer, while there is insufficient evidence for an increased risk for breast cancer development. In contrast, *BARD1* and *PALB2* mutation carriers have an increased risk for breast cancer development without an associated increased risk for tubo-ovarian cancer (25).

A number of morphological features perceived at the time of microscopic examination have been described in *BRCA*-related breast and tubo-ovarian cancers, and the discussion of these characteristic features and their clinical relevance will be the main topic of this review. In addition, tumor and germline genetic testing will also be discussed.

## HBOC-Associated Breast Cancer

Breast carcinoma is the most common malignancy arising in female patients with HBOC as a result of germline *BRCA1/2* mutations. For risk reduction, bilateral mastectomy is recommended for all *BRCA1/2* mutation carriers (26). From a pathological perspective, *BRCA1* and *BRCA2*-associated breast tumors have been shown to differ on both morphological and molecular levels (Table 1). Furthermore, *BRCA1*-associated tumors tend to be more difficult to visualize on mammographic studies compared to *BRCA2*-associated tumors which more commonly present with microcalcifications and/or isolated ductal carcinoma in situ (27).

**TABLE 1:** Morphological and molecular features of *BRCA1* and *BRCA2*-associated breast cancer.

Morphological features	*BRCA1*	*BRCA2*
Tubule formation	Minimal to none, “medullary” solid growth	Abundant
Nuclear grade	High	Variable, usually low to intermediate
Mitotic rate	High	Variable
Overall Nottingham grade	High	Variable, usually grade 1 or 2
Intrinsic molecular subgroup	Basal-like	Luminal-like (luminal A)
Biomarker profile	ER-, PR-, HER2-	ER+, PR+, HER2-

*ER, estrogen receptor; PR, progesterone receptor; and HER2, human epidermal growth factor receptor 2.*

### BRCA1-Associated Breast Cancer

**FIGURE 1:**
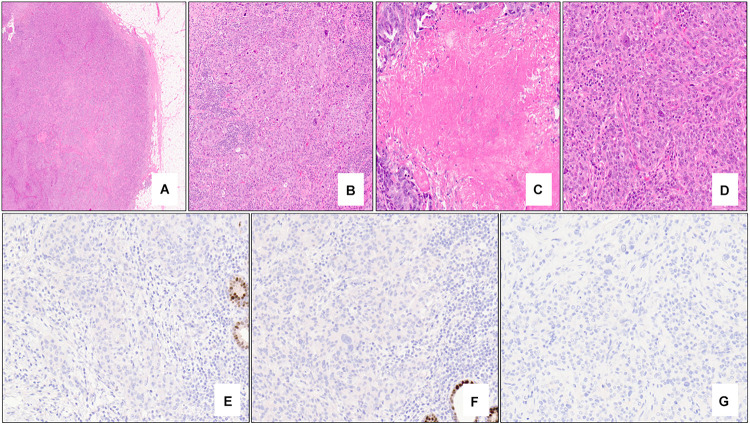
Nottingham grade 3 invasive ductal carcinoma (no special type) of the breast associated with *BRCA1* germline mutation and “triple-negative” biomarker profile. The tumor exhibits solid architecture and pushing border (**A** – 2x mag), prominent intra-tumoral lymphocytes (**B** – 10x mag), large areas of necrosis (**C** – 15x mag), and high-grade nuclear atypia with prominent nucleoli (**D** – 20x mag). The tumor is triple-negative lacking expression of estrogen receptor (**E** – 20x mag), progesterone receptor (**F** – 20x mag), and HER2 (**G** – 20x mag). Note the positive internal control cells (benign terminal duct lobular units) in **(E,F)**. **(A–D)** hematoxylin-eosin stain; **(E–G)** immunohistochemistry.

Morphologically, *BRCA1*-associated breast carcinomas are most commonly a high-grade invasive ductal carcinoma of no special type and display minimal if any tubule or glandular formation, markedly pleomorphic nuclei (significant variation in size and shape), vesicular chromatin, prominent nucleoli, and high mitotic activity. A “medullary” appearance with a sheet-like proliferation of tumor cells, pushing borders, necrosis, and prominent peri- and intra-tumoral lymphocytes has also been described (Figure 1). Of note, classical criteria for medullary carcinoma of the breast include syncytial architecture composing >75% of the tumor mass, histological circumscription with pushing margins, lack of tubular differentiation and in situ carcinoma, a prominent and diffuse lymphocytic infiltrate, and round tumor cells with abundant cytoplasm and pleomorphic high-grade vesicular nuclei containing one or several nucleoli (28, 29). Given that these diagnostic criteria are difficult to apply and lead to high interobserver variability, the World Health Organization (WHO) proposes the term “invasive carcinoma of no special type with medullary pattern” to describe a tumor exhibiting some or all of the above characteristics (30). From a molecular perspective, the majority of these *BRCA1*-associated breast tumors fall into the “basal-like” subtype of breast cancer, one of the four common intrinsic molecular subtypes (31). “Basal-like” tumors are characterized by overexpression of genes associated with basal epithelium and proliferation and minimal to no expression of genes associated with estrogen receptor (ER) and human epidermal growth factor receptor 2 (HER2). This gene expression profile is reflected in the immunohistochemical expression of basal markers including cytokeratin 5/6 and epidermal growth factor receptor (EGFR), in addition to lack of expression of ER and progesterone receptor (PR) as well as HER2 (27, 32–35). Metaplastic carcinomas have also been reported in *BRCA1* mutations carriers (36, 37).

### BRCA2-Associated Breast Cancer

**FIGURE 2:**
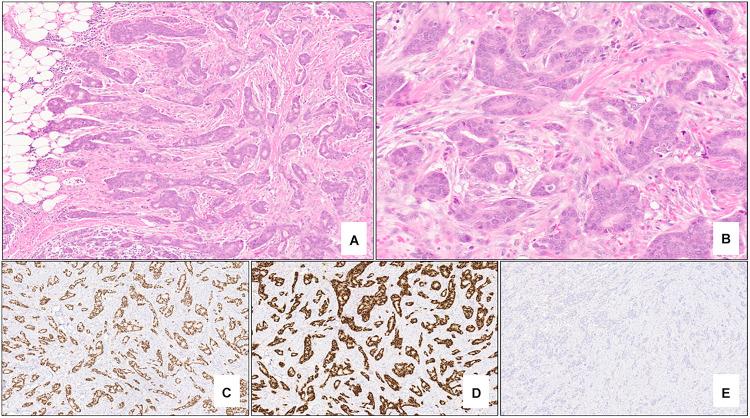
Nottingham grade 2 invasive ductal carcinoma (no special type) of the breast associated with *BRCA2* germline mutation. The tumor shows >75% tubule formation (**A** – 5x mag) with moderate nuclear pleomorphism and inconspicuous mitotic activity (**B** – 20x mag). Almost 100% of the neoplastic cells are strongly positive for estrogen receptor (**C** – 5x mag) and progesterone receptor (**D** – 5x mag), and negative (score 0) for HER2 protein overexpression (**E** – 5x mag). **(A,B)** hematoxylin-eosin stain; **(C–E)** immunohistochemistry.

In contrast to *BRCA1*-associated breast cancers, *BRCA2*-associated tumors are very similar to sporadically-occurring “luminal-type” tumors (31). This group comprises the most common of the intrinsic molecular subtypes of breast cancer (luminal A) and is characterized by variable expression of genes typically expressed in luminal breast epithelium and those associated with ER (31). Morphologically, these tumors are most commonly invasive ductal carcinoma of no special type of variable grade and do not appear to have a specific morphology, although lobular carcinomas have been reported to be more likely related to *BRCA2* mutations (32). Immunohistochemically, *BRCA2*-associated tumors are typically positive for low molecular weight keratins, ER and PR and lack HER2 protein overexpression (38) (Figure 2).

### Non-BRCA-Associated Breast Cancer

To date, no specific morphological features have been described in tumors associated with mutations in non-*BRCA* genes which impart increased risk for breast cancer development.

## HBOC-Associated Tubo-Ovarian Cancer

### General Tumor Morphology

Germline *BRCA1/2* mutations are found in approximately 15% of women with ovarian epithelial neoplasms, the most common tubo-ovarian tumor subtype (39). The hallmark histopathologic diagnosis of HBOC-related tubo-ovarian cancer due to *BRCA* mutations is that of high-grade serous carcinoma (40–42), and the frequency of *BRCA1* and *BRCA2* germline mutations increases to approximately 25% in patients diagnosed with these neoplasms (43–45). In addition to high-grade serous carcinoma, other ovarian tumor histotypes including those with endometrioid, mucinous and clear cell differentiation (and others) have also been described to varying degrees in *BRCA*-associated cohorts (32, 33, 39, 46, 47), although some of these studies did not have central review of all pathological specimens (48).

**FIGURE 3:**
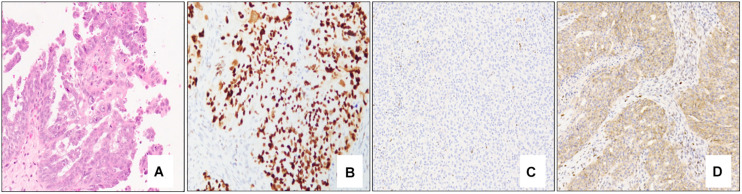
Classical high-grade serous carcinoma composed mostly of papillae lined by atypical epithelial cells with irregular and pleomorphic nuclei and prominent nucleoli (**A** – 10x mag). Immunohistochemical staining patterns of p53 in high-grade serous carcinoma: Strong, diffuse nuclear staining (**B** – 10x mag), complete absence of staining/null pattern (**C** – 10x mag), and cytoplasmic staining (**D** – 10x mag); note the positive internal control (lymphocytes) in **(C)**. **(A)** hematoxylin-eosin stain; **(B–D)** immunohistochemistry.

Morphologically, classical high-grade serous carcinoma shows expansile and infiltrative growth of glands and papillae with slit-like spaces. Tumor nuclei are generally enlarged and irregular with prominent nucleoli and brisk mitoses, including atypical forms (Figure 3A). Immunohistochemically, high-grade serous carcinomas express p53 in an aberrant pattern (most commonly either nuclear overexpression or complete absence of expression, and less commonly cytoplasmic pattern expression) (Figures 3B–D), in addition to CK7, PAX8, and WT-1. ER (and much less commonly PR) usually shows diffuse and strong expression, although staining may be variable in some cases. P16 expression is typically diffuse, strong and block-like.

### Specific Tumor Characteristics

**FIGURE 4:**
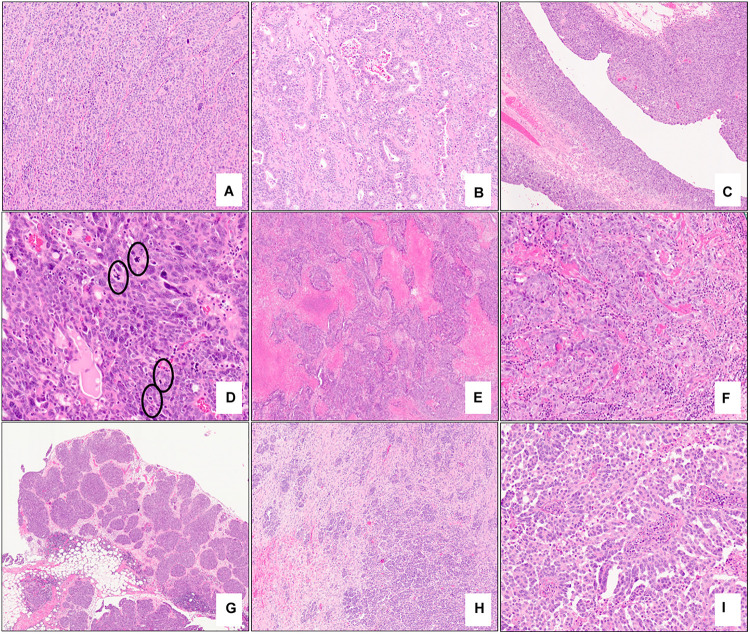
Examples of morphologic features of primary and metastatic high-grade serous carcinoma with *BRCA* mutations. Solid (**A** – 10x mag), pseudo-endometrioid (**B** – 10x mag), and transitional cell carcinoma-like (**C** – 5x mag) architectural patterns; note features reminiscent of papillary urothelial carcinoma in **(C)**. Brisk mitotic activity (**D** – 20x mag), geographic necrosis (**E** – 2x mag), and increased tumor infiltrating lymphocytes (**F** – 10x mag) are present. Omental involvement by *BRCA*-associated high-grade serous carcinoma. A well-circumscribed tumor nodule with a rounded edge and pushing border (**G** – 2x) and an infiltrative focus composed exclusively of micropapillae (**H** – 5x mag, **I** – 15x mag). **(A–I)** hematoxylin-eosin stain.

A variety of specific morphological characteristics have been described in the context of *BRCA*-associated high-grade serous carcinoma (Table 2). Fujiwara et al. showed that tubo-ovarian carcinomas in a cohort of *BRCA1* germline mutation carriers tended to exhibit high-grade and serous/undifferentiated histology, prominent tumor infiltrating lymphocytes (TILs), marked nuclear atypia with giant/bizarre forms, and abundant mitotic figures; these features had a negative predictive value of >94% and a positive predictive value of 21% for *BRCA1* germline mutation status (49). Soslow et al. studied tumors from patients with germline *BRCA1/2* mutations in addition to tumors with somatic *BRCA1/2* mutation or promoter hypermethylation and found that *BRCA1*-associated high-grade serous carcinomas exhibit high mitotic rates, increased TILs, geographic/comedo-type necrosis, and non-traditional architectural patterns including Solid, pseudo-Endometrioid, and Transitional-like (SET) features. *BRCA2*-mutated tumors also had SET features but tended to have a relative deficiency of TILs and necrosis (42). Examples of SET features are shown in Figures 4A–F; note the sheet-like growth of the solid pattern, the glandular spaces in the pseudo-endometrioid pattern, and broad and multi-layered papillary-like structures of the transitional-like pattern.

**TABLE 2:** Morphological features of *BRCA1* and *BRCA2* associated high-grade serous carcinoma.

Morphological features	*BRCA1*	*BRCA2*
Architecture	Frequent SET morphology	
Nuclear atypia	Marked	
Necrosis	Abundant	Relatively deficient
TILs	Abundant	Relatively deficient
Morphology of metastases	Pushing invasion or infiltrative invasion composed exclusively of micropapillae	
Immunophenotype	CK7 +, PAX8 +, WT-1 +, ER +, PR +/-, aberrant expression pattern of p53, and diffuse p16	

*SET, Solid, pseudo-Endometrioid, and Transitional-like; TILs, tumor infiltrating lymphocytes; CK7, cytokeratin 7; ER, estrogen receptor; and PR, progesterone receptor.*

Prior to the recognition of SET features in high-grade serous carcinoma, tumors exhibiting these morphological findings were often misdiagnosed as high-grade endometrioid, transitional cell, or undifferentiated carcinomas. In a more recent study, Ritterhouse et al. confirmed that tumors with homologous recombination deficiency, including those diagnosed in *BRCA1* and *BRCA2* mutation carriers, are six times more likely to exhibit non-classical (SET or ambiguous) features of high-grade serous carcinoma (50).

Although the aforementioned features are associated with *BRCA*-mutated tumors, the data to date have not been able to demonstrate differences to accurately distinguish tumors associated with germline mutations versus somatic mutations and *BRCA* promoter methylation based on morphology alone. As such, confirmatory genetic testing is necessary. However, identification of morphological features associated with *BRCA1/2* is useful for clinical guidance and potential genetic screening.

In addition to morphological features identified at the primary tumor site, specific architectural patterns (metastatic deposits with rounded and pushing contours/“medullary-like” invasion or infiltrative invasion composed exclusively of micropapillae) identified at metastatic sites have also been found to be highly concordant with *BRCA1/2* mutation status and display a high level of agreement among observers (kappa >0.9) (51) (Figures 4G–I). Cases which displayed those features at metastatic sites most commonly also exhibited SET features in both the metastatic and primary tumors. Distinction between these two patterns appears to be prognostically relevant as an infiltrative micropapillary pattern has been more commonly identified in metastatic tumor foci from patients who suffered recurrence or death from disease, compared to those with pushing pattern metastases (52). Interestingly, it has been hypothesized that metastatic tumor architecture may influence the ease of resection of these deposits and thus may contribute to surgeons’ ability to achieve optimal tumor debulking in these patients (51, 53).

Interestingly, loss of BRCA1 protein expression by immunohistochemistry has been shown to correlate with *BRCA1* mutation status or *BRCA1* promoter hypermethylation with negative predictive values ranging from 95% to 100% (54, 55). Despite these findings, this technique is not used in routine clinical practice, likely because of a number of limitations including internal control issues, the requirement for nuanced interpretation, and because at least some *BRCA1* clones are not helpful in detecting mutations in certain parts of the gene (54, 56). Immunohistochemistry for the assessment of BRCA2 expression also exists; however, studies to date which have evaluated its use appear to be heterogeneous and have shown mixed results (57).

### Role of the Fallopian Tube in the Pathogenesis of High-Grade Serous Carcinoma

It is now widely accepted that the majority of high-grade serous carcinomas arise from fallopian tube epithelium (58–60). Serous tubal intraepithelial carcinoma (STIC) has been recognized as an early form/precursor of high-grade serous carcinoma (61, 62). Approximately 40–60% of all women with high-grade serous carcinoma will harbor a STIC lesion (63, 64). STICs are most commonly identified in the fimbriated end of the fallopian tube near the tubal-peritoneal junction. Although precursor lesions in the fallopian tube had been described prior to the implementation of risk reducing bilateral salpingo-oophorectomy (rrBSO), the possible relationship with ovarian high-grade serous carcinoma was only noted after implementation in the management of patients with germline *BRCA1/2* mutations. Approximately 5–10% of patients with *BRCA1/2* mutations who undergo rrBSO will harbor some form of early serous neoplasia (discussed below), most commonly STIC (60, 65, 66). It should be noted that rrBSO is recommended by multiple guidelines for *BRCA1/2* mutation carriers between the ages of 35 to 40 (or once childbearing is complete or 10 years younger than the age of the youngest first degree relative diagnosed with tubo-ovarian cancer). The age of prophylactic surgery may be delayed until 40 to 45 years of age in some *BRCA2* carriers in addition to *RAD51C, RAD51D*, and *BRIP1* mutation carriers (26, 67). Salpingectomy only followed by interval oophorectomy is another therapeutic alternative being actively investigated (68).

**FIGURE 5:**
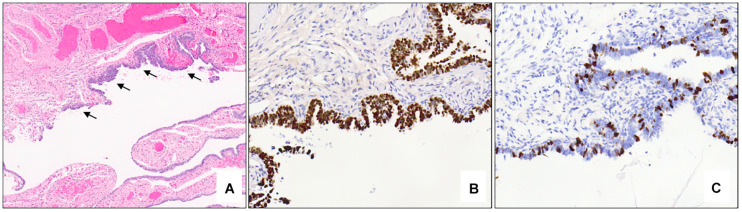
Serous tubal intraepithelial carcinoma. Low power examination of the fimbriated end of the fallopian tube shows an atypical focus (black arrows) which appears darker and crowded compared to the adjacent benign epithelium (**A** – 5x mag). Immunohistochemical stains show aberrant expression of p53 with strong, diffuse nuclear staining (**B** – 10x mag) and an elevated (>10%) Ki-67 proliferation index (**C** – 10x mag) in the atypical area. **(A)** hematoxylin-eosin stain, **(B,C)** immunohistochemistry.

Microscopically, STICs exhibit multilayered epithelium with minimal to mild tufting and stratification, loss of polarity, hyperchromatic and often pleomorphic nuclei, and prominent nucleoli; cilia are absent, and mitotic figures and apoptotic bodies are usually seen (69). A morphological and immunohistochemical algorithm was proposed in 2011 and validated in 2012 for standardization of the classification of STIC, according to which STICs should exhibit an elevated Ki-67 proliferation index (>10%) and aberrant expression of p53 protein (overexpressed in >75% of cells or completely absent/null–pattern) (Figure 5) (69, 70). Precursor lesions that do not meet the morphological and/or immunohistochemical criteria for STIC are categorized as serous tubal intraepithelial lesion (STIL) or p53 signature (70). STIL may be diagnosed in a number of different scenarios: (a) tubal epithelium with unequivocal features of STIC and aberrant p53 expression and a low (<10%) Ki-67 proliferation index, or wild-type p53 expression and high (>10%) Ki-67 index, or wild-type p53 expression and low (<10%) Ki-67 index; (b) atypical tubal epithelium suspicious for STIC and either aberrant p53 expression and low (<10%) proliferation index, or wild-type p53 expression and high (>10%) Ki-67 index; and (c) morphologically normal epithelium with aberrant p53 expression and a high (>10%) Ki-67 index. P53 signature is defined by morphologically normal (or near normal) epithelium with aberrant p53 expression and a low (<10%) Ki-67 proliferation index. These lesions have been shown to share *TP53* mutations with adjacent invasive carcinomas (64, 71, 72).

An alternate classification scheme exists which does not rely on Ki-67 proliferation index and which rather focuses on epithelial atypia combined with aberrant p53 immunohistochemical staining. Some forms of benign epithelial atypia (i.e., secretory or stem cell outgrowths, i.e., SCOUTs) are discrete proliferations which may lose cilia but lack aberrant p53 immunohistochemical expression. When aberrant p53 staining is detected in a discretely altered epithelium lacking cilia, the lesion may be classified one of three ways: (a) as a STIC when a loss of polarity is detected, (b) as a serous tubal epithelial proliferation/lesion of uncertain significance when polarity is retained but atypia is present, or (c) benign serous tubal intraepithelial proliferation/p53 signature when polarity is retained and atypia is absent (73).

It should be noted that there is still considerable work being done with regard to the origin of tubo-ovarian high-grade serous carcinoma as in some patients (especially those diagnosed with high stage tumors), no evident STIC is ever identified. In particular, tumors exhibiting SET morphology have a lower level of correlation with the presence of STIC compared to tumors with classical morphology (74, 75). This finding suggests that tumors with SET morphology may derive from a number of different mechanisms including rapid overgrowth of STIC or an alternate tubal precursor lesion (75). This has led to some investigators to question whether the carcinogenic sequence leading to high-grade serous carcinoma is more complex (76). Currently, assignment of the fallopian tube as a primary site is based on the finding of STIC, invasive mucosal carcinoma with or without STIC, or if the fallopian tube is partially or entirely incorporated into tubo-ovarian mass. Tumors lacking STIC or invasive mucosal carcinoma in either fallopian tube in presence of macroscopic or microscopic ovarian involvement can be classified as primary ovarian regardless of presence and size of peritoneal disease (77). High-grade serous carcinoma can be classified as primary peritoneal if both fallopian tubes and ovaries have been examined entirely, and are macroscopically and microscopically normal.

### Non-BRCA-Associated Tubo-Ovarian Cancer

Similar to breast cancers associated with mutations of non-*BRCA* genes involved in homologous recombination, limited data currently exists which definitively describes any specific morphological features associated with these tumors.

## Additional Tumor Considerations

Although the prototypical female cancers associated with HBOC are those arising in the breast and ovary/fallopian tube/peritoneum, recent work suggests that some endometrial carcinomas may be associated with underlying BRCA alterations. de Jonge et al. have shown homologous recombination deficiency in 24% of endometrial cancers, all with non-endometrioid morphology (78). In addition, endometrial carcinomas in germline *BRCA1* or *BRCA2* mutation carriers have also been reported to be of non-endometrioid subtype in 58% of cases and grade 3 histology in 79% of cases, and most commonly fall into the *TP53*-mutated molecular subgroup defined by The Cancer Genome Atlas Research Network (TCGA) in 92% of cases (79). Overall, these interesting findings warrant additional studies to establish whether endometrial cancer patients may benefit from treatment targeting homologous recombination deficiency. The findings also raise important potential consequences from counseling/surveillance perspectives.

Besides malignancies arising in the breast and gynecological organs, an increased risk of other neoplasms, including pancreatic carcinoma, gastric carcinoma and cutaneous malignant melanoma (80–82), has been reported in HBOC patients, particularly in individuals with germline *BRCA2* mutations. However, in contrast to the better characterized phenotype-genotype correlations in female breast and gynecological tumors discussed above, no particular *BRCA*-associated morphological features have been described in these tumors, to the best of our knowledge. Nevertheless, awareness of the potential association that these tumors may have with an underlying *BRCA* mutation is very important, especially when they are identified in a patient without a known family history.

## Clinical Implications

### Pathological Processing

The concept that the many high-grade serous carcinomas arise from the fallopian tube has played a major role in driving the evolution of how prophylactic surgical specimens from HBOC patients are evaluated. Currently, it is standard practice to examine these specimens according to the SEE-FIM (Sectioning and Extensively Examining the Fimbriated end of the fallopian tube) protocol (60) which dictates that the distal 2 cm of each fimbriated end should be sectioned at 2 mm intervals along the long axis and entirely submitted for microscopic examination. The remainder of the fallopian tube is also to be sectioned at 2 mm intervals and entirely submitted, in addition to both ovaries (in the absence of any grossly evident lesion). The purpose of the SEE-FIM protocol is to maximally expose tubal fimbrial epithelium for microscopic evaluation, as STIC lesions are typically focal and not grossly evident.

### Treatment

Importantly, the identification of an germline-associated *BRCA1/2*-mutated tumor indicates not only an underlying germline defect (in the patient and perhaps also in her family members), but also implies certain important prognostic and treatment connotations. For example, mutations involving genes whose protein products are involved in homologous recombination have been shown to be associated with chemotherapeutic platinum sensitivity and improved survival in both breast and tubo-ovarian cancer patients (46, 50, 83). Similarly, triple-negative breast cancer patients harboring defects in homologous recombination proteins have been shown to exhibit increased sensitivity to both platinum-based and standard chemotherapy regimens (84, 85), although the effect on prognosis is more complex (86). The underlying molecular abnormalities due to homologous recombination deficiency indicate that these malignancies can be treated with novel poly ADP-ribose polymerase (PARP) inhibitors which act to limit repair of single strand breaks (87) and thus lead to tumor cell death due to the overwhelming genetic instability (88). Recent studies have also shown that *BRCA*-deficient tumors have elevated expression levels of programmed cell death protein 1 (PD-1) and programmed cell death ligand 1 (PD-L1) in tumor-associated immune cells, indicating that checkpoint inhibitors may be useful in the treatment of *BRCA*-associated cancers (89–91).

### Genetic Testing

Tumor and germline genetic testing is variably performed in patients affected by breast (92) and ovarian carcinomas (93). It has been shown that triaging women for genetic testing based on family history alone will miss up to 30% of affected individuals (94). Nevertheless, genetic testing for breast cancer patients is still largely based on family history risk (Table 3) (26). However, it has been suggested that all patients with breast cancer should undergo genetic testing in order to optimize treatment and improve survival, but also to mitigate risk for family members that are healthy mutation carriers (95).

**TABLE 3:** Criteria for breast and/or ovarian cancer genetic assessment.

Any individual (at any age):	• With a known pathogenic or likely pathogenic variant in a cancer susceptibility gene within the family • With a known pathogenic or likely pathogenic variant in a cancer susceptibility gene discovered on tumor testing
Any individual (at any age) who develops the following:	• Ovarian cancer • Pancreatic cancer • Metastatic prostate cancer • Is of Ashkenazi Jewish ancestry and develops breast cancer or high-grade prostate cancer
Any individual with breast cancer and the following:	• Diagnosis is at ≤50 years of age • Development of a triple-negative cancer at ≤60 years of age • Two separate breast cancers (either in the same or contralateral breast, synchronous or metachronous) •≥2 close blood relatives diagnosed with breast cancer at any age
Any individual with breast cancer at any age and ≥1 close blood relative with the following:	• A diagnosis of breast cancer at ≤50 years of age • Ovarian cancer • Male breast cancer • Pancreatic cancer • High-grade or metastatic prostate cancer
Any individual who does not meet the above criteria but who has a first or second degree relative with any of the following:	• Diagnosis of breast cancer at ≤45 years of age • Ovarian cancer • Male breast cancer • Pancreatic cancer • Metastatic prostate cancer •≥2 separate breast cancers in a single individual •≥2 individuals with breast cancer on the same side of the family with at least one diagnosed ≤50 years of age
Any individual with a personal and/or family history on the same side of the family of ≥3 of a variety of malignant neoplasms	

*A close blood relative includes a first, second, or third degree relative.*

For tubo-ovarian cancer, an approach driven by histological tumor features has been adopted in a number of institutions given the strong association between high-grade serous morphology and *BRCA1/2* mutations. It is generally recommended that every tubo-ovarian/primary peritoneal high-grade serous carcinoma be tested for at least somatic *BRCA1/2* gene mutations, in addition to mutations in other high-risk genes (96). At some institutions, this testing is done reflexively once a diagnosis of high-grade serous carcinoma has been made. Diagnostic accuracy is therefore critical. Importantly, mutation testing should be done regardless of the presence or absence of the morphological features discussed above. Some organizations have recommended germline testing in all patients with invasive non-mucinous epithelial ovarian, fallopian tube or peritoneal cancers (i.e., not only high-grade serous carcinomas, but also endometrioid, clear cell, and seromucinous subtypes) (97–99). If tumor testing is undertaken and a mutation is identified, referral to genetic counseling for consideration of additional germline testing is necessary (if not already done) as a proportion of pathogenic mutations identified in tumor tissue will be of germline origin. A number of genetics referral models exist with each model having its advantages and disadvantages (100).

In the past, single gene testing was used for the purpose of assessing underlying mutations. However, the use of comprehensive multigene panel testing has become increasingly prevalent and has helped to identify additional patients with *BRCA1/2* mutations as well as patients with mutations in other genes associated with an increased risk. Although a multigene testing approach provides advantages in terms of comprehensive assessment, cost and turnaround time, the goals of practical clinical utility and ease of interpretation should always be kept in mind (101, 102), in addition to the care that should be taken to ensure extensive and in-depth clinical and analytical validation (103).

## Conclusion

In this review, we have discussed the HBOC syndrome from a pathological perspective and have described specific characteristics of *BRCA1* and *BRCA2-*associated breast and tubo-ovarian neoplasms. Pathologists play a critical role in the identification and triage of affected patients, particularly those without a known family history, as a number of morphological features associated with these *BRCA*-mutated tumors have been reproducibly described and are easily recognized. Accurate and timely pathological assessment and interpretation is critical given the implications for prognosis, therapy and genetic testing. Ongoing research will continue to refine our understanding of HBOC syndrome pathology, including how non-*BRCA* gene mutations affect tumor morphology, behavior and prognosis. In addition, our understanding will continue to develop regarding precursor lesions of high-grade serous carcinoma and other neoplasms arising in the context of the syndrome, including endometrial carcinoma and other non-gynecologic tract tumors.

## Data Availability Statement

The original contributions presented in the study are included in the article/supplementary material, further inquiries can be directed to the corresponding author.

## Author Contributions

AH and GT contributed equally to the conception, initial drafting, and final editing of this review article. Both authors approved the submitted version.

## Conflict of Interest

The authors declare that the research was conducted in the absence of any commercial or financial relationships that could be construed as a potential conflict of interest.
